# Thyroid Function Before, During, and After COVID-19

**DOI:** 10.1210/clinem/dgaa830

**Published:** 2020-11-12

**Authors:** Bernard Khoo, Tricia Tan, Sophie A Clarke, Edouard G Mills, Bijal Patel, Manish Modi, Maria Phylactou, Pei Chia Eng, Layla Thurston, Emma C Alexander, Karim Meeran, Alexander N Comninos, Ali Abbara, Waljit S Dhillo

**Affiliations:** 1 Department of Endocrinology, Division of Medicine, Royal Free Campus, University College London, London, UK; 2 Division of Diabetes, Endocrinology and Metabolism, Department of Metabolism, Digestion and Reproduction, Imperial College London, London, UK; 3 Department of Endocrinology, Imperial College Healthcare NHS Trust, London, UK

**Keywords:** COVID-19, SARS-CoV-2, thyroid function, thyroid gland

## Abstract

**Context:**

The effects of COVID-19 on the thyroid axis remain uncertain. Recent evidence has been conflicting, with both thyrotoxicosis and suppression of thyroid function reported.

**Objective:**

We aimed to detail the acute effects of COVID-19 on thyroid function and determine if these effects persisted on recovery from COVID-19.

**Design:**

A cohort observational study was conducted.

**Participants and Setting:**

Adult patients admitted to Imperial College Healthcare National Health Service Trust, London, UK, with suspected COVID-19 between March 9 to April 22, 2020, were included, excluding those with preexisting thyroid disease and those missing either free thyroxine (FT4) or thyrotropin (TSH) measurements. Of 456 patients, 334 had COVID-19 and 122 did not.

**Main Outcome Measures:**

TSH and FT4 measurements were recorded at admission, and where available, in 2019 and at COVID-19 follow-up.

**Results:**

Most patients (86.6%) presenting with COVID-19 were euthyroid, with none presenting with overt thyrotoxicosis. Patients with COVID-19 had a lower admission TSH and FT4 compared to those without COVID-19. In the COVID-19 patients with matching baseline thyroid function tests from 2019 (n = 185 for TSH and 104 for FT4), TSH and FT4 both were reduced at admission compared to baseline. In a complete case analysis of COVID-19 patients with TSH measurements at follow-up, admission, and baseline (n = 55), TSH was seen to recover to baseline at follow-up.

**Conclusions:**

Most patients with COVID-19 present with euthyroidism. We observed mild reductions in TSH and FT4 in keeping with a nonthyroidal illness syndrome. Furthermore, in survivors of COVID-19, thyroid function tests at follow-up returned to baseline.

The coronavirus disease 2019 (COVID-19) pandemic continues to affect the global community, and as understanding of its pathophysiology deepens, so too does interest in the endocrine effects of its causative coronavirus, SARS-CoV-2. Coronaviruses are known to have direct effects on several endocrine glands, including the thyroid gland. In patients infected with SARS-CoV, a related coronavirus to SARS-CoV-2, damage to the follicular and parafollicular cells of the thyroid was demonstrated at post-mortem (1). Additionally, coronaviruses have been detected in the pituitary gland post mortem (2), and reduced staining for thyrotropin (TSH) has been observed in the anterior pituitary gland of patients infected with SARS-CoV (3). Furthermore, SARS-CoV-2 enters cells using the angiotensin-converting enzyme 2 (ACE2) receptor, which is highly expressed in the thyroid gland (4). Thus, the hypothalamic-pituitary-thyroid axis may be susceptible to disturbance in patients with COVID-19. Currently there is contradictory evidence surrounding the effect of COVID-19 on thyroid function. Subacute thyroiditis presenting with overt thyrotoxicosis has been reported with COVID-19 (5-7). On the other hand, in a study undertaken in China of 50 patients with COVID-19, Chen et al (8) observed a generalized reduction in TSH, total thyroxine (T4), and tri-iodothyronine (T3) more consistent with a nonthyroidal illness pattern.

Although these studies provide useful information, they do have limitations. They have included incomplete data, for example, measuring thyroid hormones only when TSH is out of range (6, 7), or have failed to exclude confounding factors such as intercurrent steroid treatment (8). There is also limited knowledge of thyroid function in patients who have recovered from COVID-19. Given these conflicting results, we undertook a large cohort observational study to understand whether thyroid gland dysfunction is a frequent feature of patients with COVID-19.

## Materials and Methods

### Ethical approval

This study was approved by the Imperial College London and Imperial College Healthcare NHS Trust governance team, which confirmed that because we are reporting on routinely collected nonidentifiable clinical audit data, no approval from a research ethics committee was additionally required under the UK Policy Framework for Health and Social Care.

### Study participants

All patients age 18 years or older admitted to Imperial College Healthcare NHS Trust across 3 teaching hospitals, St Mary’s, Charing Cross, and Hammersmith, London, United Kingdom, with a clinical suspicion of COVID-19 between March 9 and April 22, 2020, were included in this observational cohort study. Patients presenting with suspected COVID-19 underwent a standard set of blood tests, including full blood count, renal function, albumin, C-reactive protein (CRP), cortisol, and thyroid function. Clinical and demographic data were extracted from patient records. Preselected demographics and comorbidities of interest (age, sex, history of diabetes, hypertension, chronic kidney disease, cardiovascular disease, endocrine disease, current diagnosis of cancer at time of admission, obstructive pulmonary disease including asthma and chronic obstructive pulmonary disease, current pregnancy) were recorded. A COVID-19 diagnosis was defined as a real-time reverse transcriptase polymerase chain reaction confirmation of infection from a nasopharyngeal swab (9). The COVID-19–negative patients were defined as those with clinical features inconsistent with COVID-19, a negative result from swabbing, and diagnosed with alternative conditions other than COVID-19. The first thyroid test from each admission episode was used for analysis. Cases were excluded from analysis if the measurement was not taken within 48 hours of admission or of diagnosis of COVID-19, if patients were documented with a previous history of thyroid disease and/or were taking thyroid hormones or antithyroid medications, if there were missing values of either free T4 (FT4) or TSH, or if they were taking glucocorticoids prior to admission. Mortality outcomes were recorded as of a database lock date of May 8, 2020. For longitudinal analysis of thyroid function tests, we searched the records of the patients included in the analysis for any thyroid function tests (FT4 or TSH) in 2019, prior to any cases of COVID-19 in the United Kingdom (“Base 2019”), and for any follow-up tests after their index admission for COVID-19 up to September 1, 2020 (“follow-up”).

### Assay methodology

All analytes were measured at North West London Pathology, a UK Accreditation Service–accredited laboratory, using Abbott Alinity series analyzers. Cortisol, FT4, and TSH were measured by an Abbott Alinity ci-series analyzer using chemiluminescent microparticle immunoassays. The precision of the cortisol assay was 10% or less total coefficient of variation (CV) for serum samples with cortisol between 83 nmol/L and 966 nmol/L, and the lower limit of detection was 22 nmol/L. The cross-reactivity of cortisone in this assay is minimal (2.7% at 1000 µg/dL). FT4 and TSH assays are both 2-step assays. The intralaboratory precision of the TSH assay was 2.1% or less CV when tested at 0.09 to 30 mU/L. The lower limit of reporting is set at 0.01 mU/L (readings of < 0.01 were set at 0.01 for data analysis). The cross-reactivity of follicle-stimulating hormone, luteinizing hormone, and β-human chorionic gonadotropin is 10% or less at up to 500 U/L, 500 U/L, and 200 000 U/L, respectively. The intralaboratory precision of the FT4 assay was 3% or less CV when tested at levels of 8.5 to 33.6 pmol/L. The lower limit of detection and reporting is 5.4 pmol/L (if reported, readings of < 5.4 were set at 5.4 for data analysis). The cross-reactivity of free 3,5,3′-triiodothyronine (FT3) is 0.0035% or less at up to 18 433 pmol/L.

### Statistical analysis

Data processing and statistical analysis were conducted using R 4.0.3 (R Foundation for Statistical Computing) and the packages “tidyverse 1.3.0,” “survival 3.2-7,” “ggpubr 0.4.0,” “rstatix 0.6.0,” and “moments 0.14.” Data distribution was assessed using density and Q-Q plots, and a D’Agostino and Pearson test where required. Wherever appropriate, a log2 transformation was used to transform skewed distributions of measured parameters (eg, of TSH, CRP, cortisol). Parametric data are presented as mean ± SD, whereas nonparametric data are presented as median with interquartile range (IQR). A *P* value of less than .05 was regarded as statistically significant. An unpaired, 2-tailed *t* test was used to compare parametric continuous variables and a Wilcoxon rank sum test for nonparametric continuous variables. For comparison of longitudinal data, a repeated-measures one-way analysis of variance for parametric data or a Friedman test for nonparametric data was used. Wilcoxon paired rank sum tests with Bonferroni adjustment were used for pairwise comparison of TSH in the longitudinal data set. A Fisher exact test was used to test for differences in categorical distribution.

## Results

### Baseline characteristics

A total of 621 patients were admitted and had thyroid function tests at admission. Of the remaining 456 patients after exclusions, 334 (73.2%) were diagnosed with COVID-19 and 122 (26.8%) were not (Table 1). Mean (SD) age in the COVID-19 group was 66.1 years (16.0 years) and in the COVID-19–negative group was 63.8 years (19.3 years). A male preponderance was observed both in the COVID-19 group (60.8%) and the control group (55.7%). By May 8, 2020, 40 (12.0%) patients with COVID-19 were admitted to intensive therapy units (ITUs), and 95 (28.4%) patients had died, significantly higher than in the COVID-19–negative group.

**Table 1.** Characteristics of patients (n = 456) diagnosed with COVID-19 and without COVID-19

COVID-19 status		No	Yes
No., %		122 (24.2)	334 (73.2)
Age, y		63.8 (19.3%)	66.1 (16.0%)
Age (stratified), y	< 45	25 (20.5%)	28 (8.4%)
	45-59	20 (16.4%)	92(27.5%)
	60-74	30 (24.6%)	84 (25.1%)
	≥ 75	47 (38.5%)	130 (38.9%)
Weight, kg		73.1 (19.1)	79.9 (22.2)^*b*^
Sex	Male	68 (55.7%)	203 (60.8%)
	Female	54 (44.3%)	131 (39.2%)
Diabetes		30 (24.4%)	132 (39.5%)^*e*^
Hypertension		49 (40.2%)	162 (48.5%)
Current diagnosis of cancer		19 (15.6%)	29 (8.7%)^*d*^
COPD/asthma		25 (20.5%)	58 (17.4%)
Chronic kidney disease		17 (13.9%)	44 (13.2%)
Cardiovascular disease		33 (27.0%)	79 (23.7%)
Pregnancy		0	2 (0.6%)
Endocrine disease other than thyroid		2 (1.6%)	17 (5.1%)
Deaths to May 8, 2020		9 (7.4%)	95 (28.4%)^*f*^
ITU admissions to May 8, 2020		3 (2.5%)	40 (12.0%)^*e*^
Free T4, pmol/L		13.11 (2.33)	12.60 (2.18)^*a*^
TSH, mU/L		1.48 (0.79-2.18)	1.03 (0.62-1.71)^*g*^
Cortisol, nmol/L		537 (380-708)	620 (454-849)^*h*^
CRP, mg/L		39 (8-125)	115 (58-175)^*i*^
Albumin, g/L		32.9 (6.8)	30.3 (5.2)^*c*^

Categorical data shown as number (percentage). Continuous variables displayed as mean (SD) if parametric or median (interquartile range) if nonparametric.

Abbreviations: COPD, chronic obstructive pulmonary disease; COVID-19, coronavirus disease 2019; CRP, C-reactive protein; ITU, intensive therapy unit; T4, thyroxine; TSH, thyrotropin.

^*a*^ *P* less than .05.

^*b*^ *P* less than .01.

^*c*^ *P* less than .001 (2-tailed unpaired t test).

^*d*^ *P* less than .05.

^*e*^ *P* less than .01.

^*f*^ *P* less than .001 (Fisher exact test).

^*g*^ *P* less than .05.

^*h*^ *P* less than .01.

^*i*^ *P* less than .001 (Wilcoxon rank sum test).

### Most patients admitted with coronavirus disease 2019 are euthyroid

We classified patients into diagnostic categories according to their FT4 and TSH values (Table 2). Most COVID-19 patients were euthyroid (86.5%); none were overtly hyperthyroid (as defined by a low TSH and high FT4) and only a small number had overt hypothyroidism (0.6%). The proportions of patients in each category did not significantly differ between COVID-19–positive and –negative patients, nor did they significantly differ between COVID-19 survivors and nonsurvivors, and patients admitted to ITU vs those not admitted to ITU with COVID-19.

**Table 2.** Thyroid diagnostic categories in patients diagnosed with COVID-19 and without COVID-19

	FT4, pmol/L	TSH, mU/L	All	COVID-19 neg	COVID-19 pos				
	9.0-23.0	0.30-4.20			COVID-19 pos	Surv	Nonsurv	Not ITU adm	ITU adm
Euthyr	↔	↔	395 (86.6%)	106 (86.9%)	289 (86.5%)	211 (88.3%)	78 (82.1%)	258 (87.8%)	31 (77.5%)
Hyper	↑	↓	0	0	0	0	0	0	0
Hypo	↓	↑	2 (0.4%)	0	2 (0.6%)	2 (0.8%)	0	2 (0.7%)	0
SC hyper	↔	↓	26 (5.7%)	8 (6.6%)	18 (5.4%)	12 (5.0%)	6 (6.3%)	13 (4.4%)	5 (12.5%)
SC hypo	↔	↑	24 (5.3%)	7 (5.7%)	17 (5.1%)	9 (3.8%)	8 (8.4%)	15 (5.1%)	2 (5.0%)
Sec hyper	↑	↔	0	0	0	0	0	0	0
Sec hypo	↓	↔	9 (2.0%)	1 (0.8%)	8 (2.4%)	5 (2.1%)	3 (3.2%)	6 (2.0%)	2 (5.0%)
*P*				.826		.337		.129	

Patients were classified into diagnostic categories according to the pattern of results falling below, within, or above the indicated reference ranges. *P* values calculated using Fisher exact test for comparison between COVID-19–negative and –positive patients, COVID-19–positive survivors and nonsurvivors, and COVID-19 patients admitted and not admitted to ITU are shown.

Abbreviations: adm, admitted; COVID-19, coronavirus disease 2019; Euthyr, euthyroid; FT4, free thyroxine; Hyper, hyperthyroid; Hypo, hypothyroid; ITU, intensive therapy unit; neg, negative; pos, positive; SC, subclinical; Sec, secondary; Surv, survivor; Nonsurv, nonsurvivor; TSH, thyrotropin.

### Admission with coronavirus disease 2019 is associated with a lower thyrotropin and free thyroxine

**Figure 1.**
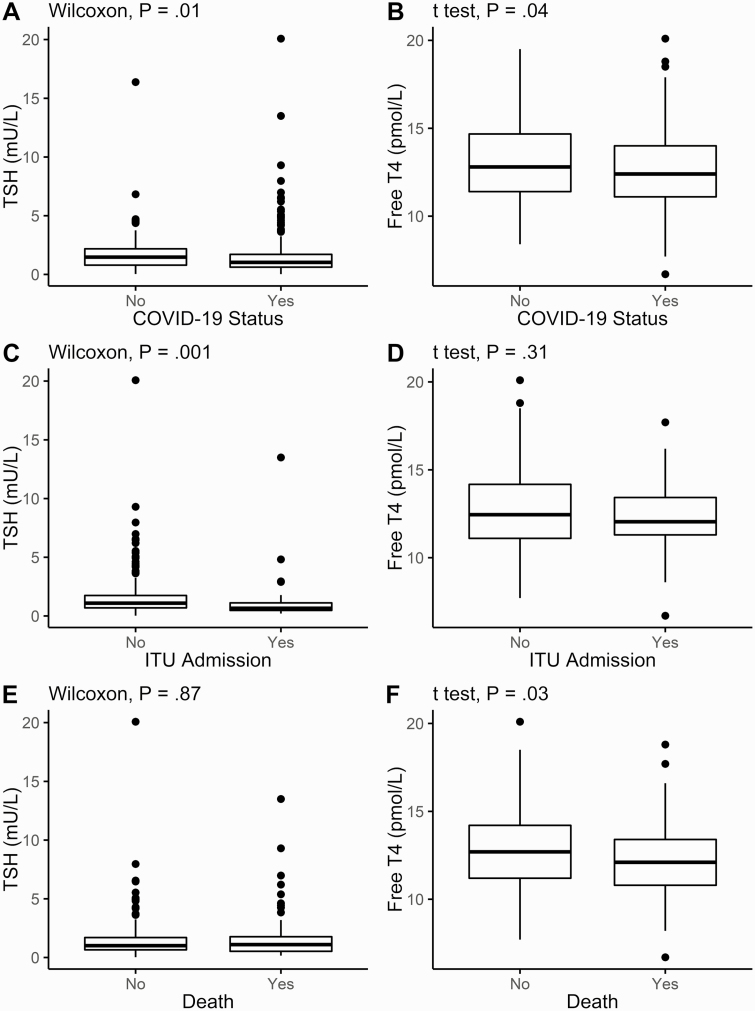
Thyrotropin (TSH) and free thyroxine (T4) on admission to the hospital. TSH and free T4 plotted with box indicating 25th and 75th percentiles, whiskers indicating fifth and 95th percentiles, and line in box indicating median, A and B, in all patients, classified by diagnosis of coronavirus disease 2019 (COVID-19); C and D, in COVID-19–positive patients, classified by admission to intensive therapy unit; E and F, in COVID-19–positive patients, classified by survival to May 8, 2020. Wilcoxon rank-sum tests used for comparison of TSH, unpaired *t* test for FT4.

Patients with COVID-19 had lower TSH with a median (IQR) of 1.03 mU/L (range, 0.62-1.71 mU/L) vs those without COVID-19: 1.48 mU/L (range, 0.79-2.18 mU/L) (Wilcoxon rank-sum test *P* = .01). Patients with COVID-19 also had a lower mean (SD) FT4 at 12.60 pmol/L (2.18 pmol/L) vs those without COVID-19 at 13.11 pmol/L (2.33 pmol/L), *P* = .04 (Fig. 1A and 1B).

Because the intraindividual variation in “setpoint” FT4 and TSH is narrower than the population reference range, we examined the subset of COVID-19 patients who had had thyroid function tests in 2019 (“Base 2019”). A total of 185 patients had a “Base 2019” median (IQR) TSH of 1.59 mU/L (1.03-2.24 mU/L) vs 1.02 mU/L (0.6-1.65 mU/L) at admission (paired Wilcoxon signed rank test *P* < .001). Of 104 patients with “Base 2019” FT4 measurements, the mean (SD) was 12.94 pmol/L (2.77 pmol/L) in 2019 vs 12.23 pmol/L (2.14 pmol/L) at admission (paired *t* test *P* = .015). In comparison, matched TSH and FT4 levels for patients without COVID-19 showed no significant difference between “Base 2019” and admission measurements (n = 62, *P* = .72, and n = 33, *P* = .74, respectively). In other words, patients admitted with COVID-19 demonstrated reductions in TSH and FT4 levels compared to “Base 2019” measurements. This phenomenon was not observed in COVID-19–negative patients.

In patients with COVID-19, those admitted to ITU had lower median TSH at 0.66 mU/L (range, 0.48-1.12 mU/L) vs 1.10 mU/L (range, 0.69-1.75 mU/L) (*P* = .001), although mean FT4 was not significantly different (Fig. 1C and 1D). Interestingly, in COVID-19 survivors, the FT4 was slightly higher at 12.76 pmol/L (2.18 pmol/L) vs nonsurvivors at 12.19 pmol/L (2.14 pmol/L) (unpaired *t* test *P* = .03), although there was no significant difference in TSH (Fig. 1E and 1F). A Cox proportional hazards analysis for survival did not discern any significant univariable relationship of FT4 nor TSH to survival.

**Figure 2.**
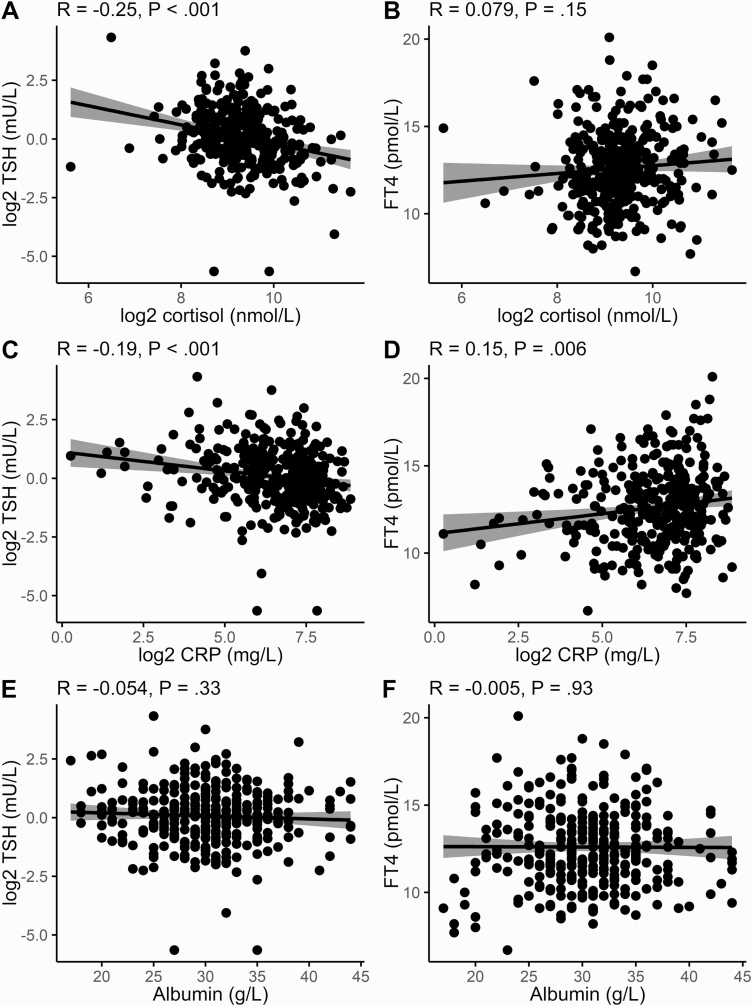
Correlations of free thyroxine (T4) and log-transformed thyrotropin (TSH) with cortisol, C-reactive protein (CRP), and albumin in coronavirus disease 2019 (COVID-19)–positive patients. Scatterplots of A, C, and E, log-transformed TSH, and B, D, and F, free T4 on the y axes are plotted against A and B, log2 cortisol, C and D, log2 CRP, and E and F, albumin on the x axes. Pearson correlation is shown at the top with its associated *P* value. Regression line (black line) and 95% CI (gray shading) are plotted.

There was a highly significant negative correlation between serum cortisol and log-transformed TSH (Pearson *R* = –0.25, *P* < .001), but no correlation between serum cortisol and FT4. There were also significant correlations between CRP and TSH (*R* = –0.19, *P* < .001) and CRP and FT4 (*R* = 0.15, *P* = .006). Serum albumin, which has previously been shown to be negatively associated with acute mortality from COVID-19 (10), was significantly lower in those diagnosed with COVID-19 (see Table 1) but no significant correlations of FT4 and TSH were seen with albumin (Fig. 2).

### Thyrotropin recovers to baseline on follow-up after coronavirus disease 2019

**Figure 3.**
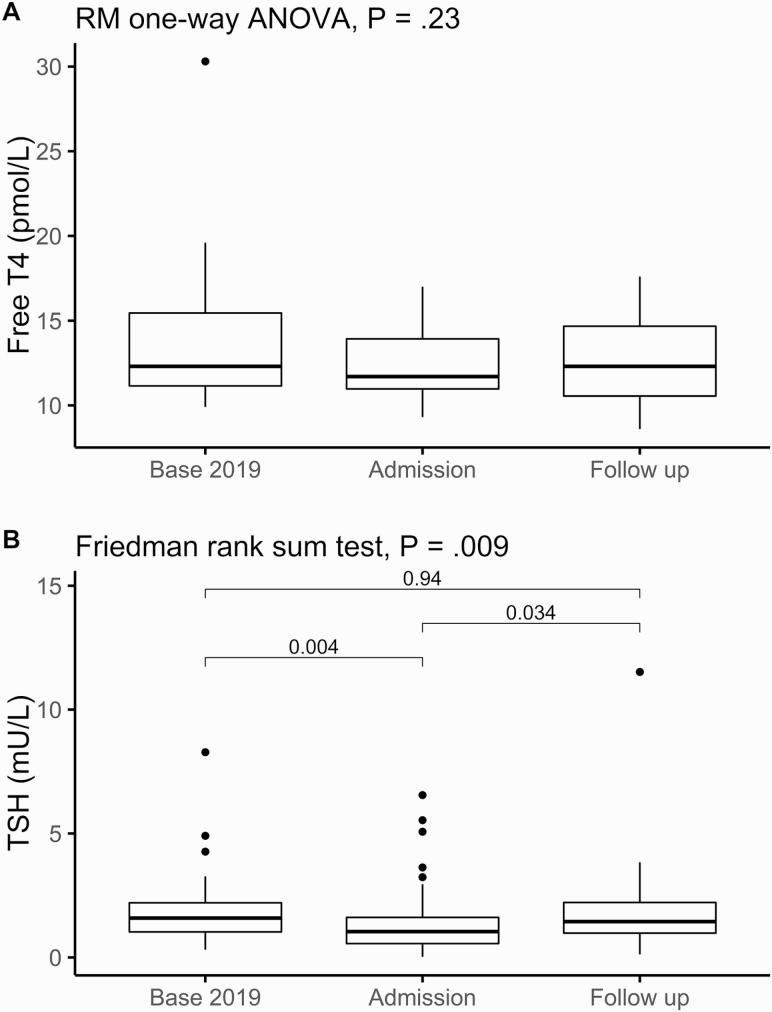
Longitudinal study of thyrotropin (TSH) and free thyroxine (T4) using 2019 baseline, admission, and follow up measurements. A (n = 20), free T4, and B (n = 50), TSH measurements, are plotted against the time point at which they were taken (baseline in 2019, admission with coronavirus disease 2019 (COVID-19), and follow-up). Repeated-measures one-way analysis of variance or Friedman rank sum test performed as indicated. Pairwise comparisons of TSH were performed with paired Wilcoxon signed-rank tests, with Bonferroni adjustment.

A small subset of COVID-19 survivors (n = 55) had follow-up thyroid function tests at a median (IQR) time since admission of 79 days (range, 52-108 days). Most of the follow-up tests (47/55) were classified euthyroid. There were only 2 cases with mildly depressed TSH and normal FT4 (“subclinical hyperthyroid”), 4 with low FT4 and normal TSH (“secondary hypothyroid”), and 2 with normal FT4 and mildly elevated TSH (“subclinical hypothyroidism”). None had overt thyrotoxicosis. In 50 patients with complete sets of TSH measurements at baseline, admission, and follow-up, the median (IQR) baseline TSH was 1.59 mU/L (range, 1.03-2.21 mU/L), at admission 1.05 mU/L (range, 0.56-1.62 mU/L), and at follow-up 1.45 mU/L (range, 0.98-2.22 mU/L) (Friedman rank sum test, *P* = .009). Pairwise comparisons showed significant differences in TSH comparing baseline vs admission (paired Wilcoxon signed rank test *P* = .004) and admission vs follow-up (*P* = .034), but baseline vs follow-up values were not significantly different (Fig. 3). In 20 patients with complete sets of FT4 measurements, the mean (SD) baseline FT4 was 14.07 pmol/L (4.74 pmol/L), at admission 12.41 pmol/L (2.00 pmol/L), and at follow-up 12.61 pmol/L (2.44 pmol/L) with no significant differences seen (repeated-measures one-way analysis of variance *P* = .23; see Fig. 3).

## Discussion

In this observational study, we investigated the acute effects of COVID-19 on thyroid function in the largest cohort of patients to date. Most patients were euthyroid at admission with COVID-19. We did however observe a small reduction in TSH and FT4 in patients with COVID-19 compared to non-COVID-19 cases. We confirmed with matched samples that there was a reduction in TSH and FT4 from 2019 baselines to admission with COVID-19, and this was not seen in patients admitted without COVID-19. Muller et al recently reported overt thyrotoxicosis in 15% of patients admitted to a high-dependency ITU with COVID-19 (6), compared to 2% in those treated in low-intensity settings; however, this study defined thyrotoxicosis as TSH less than 0.28 mU/L and/or FT4 greater than 21.9 pmol/L, reflecting the fact that TSH was the first-line thyroid function test and FT4 was measured in only 24% of their patients. We also note that in their study, TSH was routinely measured in all high-dependency ITU patients but less frequently measured in all low-dependency ITU patients, possibly introducing bias. Lania and colleagues similarly reported overt thyrotoxicosis in 10.8% of their cohort of 287 patients with COVID-19 who were treated outside intensive care, but measured thyroid hormones in only 25% of patients (7). In contrast, our study included complete sets of FT4 and TSH measurements and the measurement of thyroid function was applied across the board as part of a standard workup. In our data set we did not see any patients with overt thyrotoxicosis (using a conservative definition of TSH < 0.30 and FT4 > 23.0), even in our 40 cases of COVID-19 admitted to the ITU. Therefore, in our cohort, there was no suggestion of a novel COVID-19–related thyroiditis/thyrotoxicosis.

The most likely explanation for the changes in thyroid function we observed is nonthyroidal illness syndrome, initially characterized by a reduction in total and FT3 and an increase in reverse T3 (rT3) in the absence of an increase in TSH; more severe or prolonged illness causes global reductions in TSH, FT4, and FT3 (11). The suppression of TSH is most likely related to elevations in proinflammatory cytokines such as interleukin-6, which are negatively correlated with TSH. An additional factor may be cortisol, which is known to suppress TSH secretion, even at physiological levels (12). Chen et al were unable to exclude exogenous glucocorticoids as a factor influencing TSH (8). No patient in our cohort received exogenous steroids, and we excluded any patients taking steroids prior to admission. Therefore, the marked elevations in endogenous cortisol secretion during COVID-19 may be an additional factor suppressing TSH (13). A possible third explanation may be a direct cytopathic effect of SARS-CoV-2 on thyrotrophs as the receptor for virion binding, ACE2, is expressed in the pituitary (14). Critically, whichever of these factors are responsible for the observed drop in TSH secretion, we found that on follow-up TSH had returned to baseline, suggesting that the changes are reversible with recovery from COVID-19.

To our knowledge, this is the largest cohort of such patients with COVID-19 to have had assessment of thyroid function at presentation. Additionally, we have uniquely presented longitudinal data gathered both prior to the COVID-19 admission and at follow-up. Overt thyroid dysfunction is not characteristically observed in most patients presenting acutely with COVID-19 nor at follow-up in survivors. Although we did see a statistically significant reduction in FT4 and TSH between baseline and admission with COVID-19, the magnitude of the reduction was small and not likely to justify treatment. These data are limited by the study’s single-center design, the absence of FT3 and rT3 measurement, and characterization of thyroid autoantibody status. However, we believe our data are sufficient to suggest that routine measurement of thyroid function in COVID-19 may not be necessary unless there are other specific indicators of thyroid disease. Further longitudinal studies including FT3 and rT3 measurement are now necessary to determine the full impact of COVID-19 on the hypothalamic-pituitary-thyroid axis.

## Data Availability

An anonymized data set and data analysis code is available on application to the corresponding author.

## Abbreviations

ACE2: angiotensin-converting enzyme 2
COVID-19: coronavirus disease 2019
CRP: C-reactive protein
FT3: free 3,5,3′-triiodothyronine
FT4: free thyroxine
ITU: intensive therapy unit
IQR: interquartile range
rT3: reverse T3
T4: thyroxine
TSH: thyrotropin.

## Additional Information

***Disclosure Summary:*** The authors have nothing to disclose.
